# A New Microstructural Approach to the Strength of an Explosion Weld

**DOI:** 10.3390/ma15227878

**Published:** 2022-11-08

**Authors:** Alexander G. Kolpakov, Sergei I. Rakin

**Affiliations:** 1 SysAn (System Analysis in Engineering), 630075 Novosibirsk, Russia; 2 Mathematics Department, Siberian Transport University, 630049 Novosibirsk, Russia

**Keywords:** local stress–strain state, explosion weld, two scale method

## Abstract

In this paper, the local stress–strain state in an explosion weld was investigated and the local strength of the welded materials near the weld analyzed. It follows from the experimental data that the explosion weld at the microlevel looks like a wavy line. In the first approximation, this wavy line may be assumed to be periodic. We used the two-scale method to analyze the corresponding interface elasticity problem. We carried out numerical computations for three of the most referenced types of weld geometry: the symmetric wave, the asymmetric wave, and the wave with crest. We found that the wave geometry of the weld leads to increase in local stress in the weld zone. The stress concentration varied from 20% to 200% in dependence on the weld geometry and the macroscopic loading. Explosion welding is accompanied by strain hardening of the materials in the welding zone. In some cases, the strain hardening may compensate for the increasing local stress. As a result, the weld may be both stronger and weaker than the welded materials.

## 1. Introduction

An explosion weld occurs when metal plates collide due to plastic deformation in the impact zone. The first works on explosion welding were carried out in the USA and the USSR and date back to the period 1958–1963, see [1,2,3,4,5,6,7,8,9,10,11]. Interest in explosion welding continues unabated to this day.

The strength of a structure/material produced by the welding is determined by the strength of both the welded materials and the weld. Many publications indicate that the bonding strength of explosion welded metals exceeds the strength of the weakest material of the pair (see, e.g., [12]) and destruction occurs at a certain distance from the plane of the connection along the least durable metal (see [11,12,13,14]). In [13,14], it is explained by the strengthening of the material in/near the weld as a result of its plastic deformation. At the same time, some publications report destruction along the weld, see, e.g., [15]. In this paper, we investigated the local stress–strain state in an explosion weld and analyzed the local strength of the welded materials in the vicinity of the weld.

**Figure 1:**
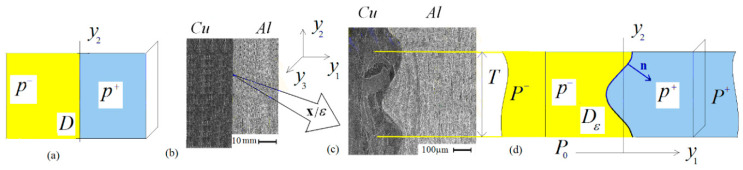
The explosion weld: (**b**) macroscopic view, (**c**) microscopic view, (**a**,**d**) models (macroscopic and microscopic).

When welding with an explosion of bodies, at the macro level, the weld looks like a straight line. However, at the micro level, the weld is wavy, see Figure 1. We call the wave-like weld and its surroundings the welding zone $P_{0}$, see Figure 1. The parts $P^{-}$ and $P^{+}$ in Figure 1d of the bodies outside the welding zone are called the main parts of the materials.

**Figure 2:**
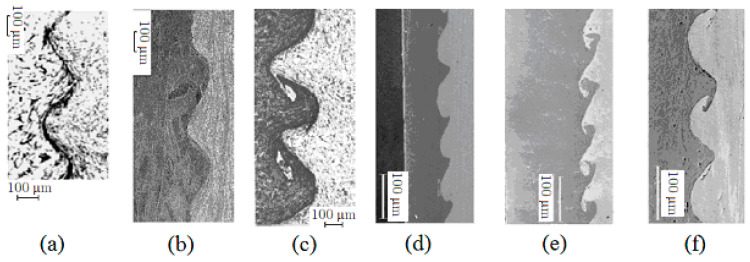
View of the weld at a microlevel: (**a**) symmetric wave; (**b**,**d**) asymmetric wave; (**c**,**e**,**f**) wave with a crest.

At a microlevel, the weld $D_{\varepsilon }$ has wavy geometry, which can be taken as periodic. The length of the period and the wave amplitude are of the order of several hundred micrometers, see Figure 2. Wave shapes vary from sinusoidal to asymmetric and to a wave with a crest (see Figure 1a–c). The wavelength and amplitude of the weld wave are of the order of hundred micrometers. These dimensions are compared with the dimension of the microstructure in metals [13]. The grain size of metals varies from 2.7 μm (0.0027mm.) to 1 mm. It is considered that large grains have a diameter from 62 μm (0.062 mm) to 1 mm, while small grains have a diameter from 2.7 μm to 44 μm, see, e.g., [14]. For example, aluminum grain diameters range from 10 μm to 100 μm. In the welds obtained by thermal welding of metals, a granular structure is different from the structure of welded metals, see [15] for details.

According to Figure 2a–c, the microstructure of the materials in an explosion weld is similar to the microstructure of the materials in the main parts of the welded materials, see Figure 2. The explosion weld differs from a fusion weld (for the fusion weld see, e.g., [13,15]).

Using the same scale in all figures in Figure 2, in Figure 2a–c (figures are taken from [16,17]), the microstructure size of the materials is compared to the wavelength of the weld. In Figure 2d–f, the microstructure size is small compared to the amplitude of the weld. In Figure 2a–c, the non-uniform microstructure is clearly seen, while in Figure 2d–f, the microstructure is practically invisible. Figure 2a displays the microstructure of the pair steel–steel, Figure 2b displays the microstructure of the pair copper–aluminum. For the materials displayed in Figure 2a–c, the first-order approximation is two homogeneous bodies connected along a wavy line (the model displayed in Figure 1c). The second-order approximation accounts for the inhomogeneous structure of the connected bodies. For the materials displayed in Figure 2d,e, the model displayed in Figure 1c may be accepted as an accurate model.

The strength of the weld is determined by the following factors:

- stress concentration caused by the wavy geometry of the joint
- change of the strength of the welded materials as a result of the intensive plastic deformation in the process of the explosion welding.

## 2. Formulation and Two-Scale Analysis of the Problem

In this paper, we analyzed in detail the local stress–strain state caused by the wavy geometry of the explosion weld. We distinguished the weld region and the main parts of the materials, which are placed far from the weld.

Although explosion welding shows a clear boundary between the welded bodies, it is impossible to separate the local stress–strain state in the weld zone $D_{\varepsilon }$ and the adjacent “left” $P^{-}$ and “right” $P^{+}$ main parts of the bodies. To describe the stress–strain state in the entire region $P^{-}\cup D_{\varepsilon }\cup P^{+}$, we used the “local perturbations” method, developed in [18,19]. The “local disturbances” are a set of functions defined in the domain $P^{-}\cup D_{\varepsilon }\cup P^{+}$ that are fast decaying with distance from the weld zone $D_{\varepsilon }$. The “local disturbances” describe both the classical boundary layers near the weld and the deformations of general form in the weld zone $D_{\varepsilon }$.

The real weld $D_{\varepsilon }$ is a wavy surface (or line, if a planar elasticity problem is considered). The characteristic dimension of the wave period and amplitude is denoted by ε. The parameter ε is small: ε≪1, and is formalized as ε→0. We assume that the weld is periodic with period εT with respect to variable $x_{2}$. Due to periodicity of the weld, the entire problem is also periodic with respect to the variable $x_{2}$ with period εT. In the “fast” variables, the problem is periodic with respect to the variable $y_{2}$ with period T (Figure 1c). We assume, in the first approximation, that the elastic constants of the materials in the welded zone are the same as in the main parts of the materials.

At the macrolevel, the weld looks like a surface (or line). We assume that this surface (line) is flat, see Figure 1a,b.

Denote x = (*s*, *t, n*) = (*x*_1_, *x*_2_, *x*_3_). the “slow” coordinates in the orthonormal coordinate system. The notation (s,t,n) is used to separate the coordinates into the “tangential” coordinates (s,t) in the plane of the weld D and is the “normal” coordinate n in the direction perpendicular to the weld, see Figure 1a. Following [20], we introduce “fast” variables $y=(y_{1},y_{2},y_{3})=x/\varepsilon$. We assume that the connecting area has a periodic structure in the variables $\left(y_{1},y_{2}\right)$ with the periodicity cell T, see Figure 2b. In the macroscopic coordinate system $\left(x_{1},x_{2},x_{3}\right)$, the periodicity cell T turns into εT.

The explosion weld is a microscopic object [21]. This means that, although the weld is small in size, it may be investigated by the methods of classical continuum mechanics [22]. The approaches based on the molecular and atomic models (see such models in [23,24,25,26]) are not intended for this paper. In this paper, we take the elasticity theory problem as the starting point of our investigation. The corresponding problem has the form:

(1) $$\left\{ \begin{array}{l} {\left(a_{ijkl}(x)u_{k,ly}\right)}_{,iy}=q_{i}(x)\;\mathrm{in}\;Q,\\ u_{}(x)=0\;\mathrm{on}\;\partial Q.\; \end{array}\right.$$

The wavy structure of the weld is taken into account by setting the local elastic constants $a_{ijkl}(x)$ in (1) in the form:

(2) $$a_{ijkl}(x)=\left\{ \begin{array}{l} a_{ijkl}^{-}\;\mathrm{on}\;\mathrm{the}\;\mathrm{left}\;\mathrm{from}\;D_{\varepsilon },\\ a_{ijkl}^{+}\;\mathrm{on}\;\mathrm{the}\;\mathrm{right}\;\mathrm{from}\;D_{\varepsilon }. \end{array}\right.$$

The local elastic constants $a_{ijkl}(x)$ are periodic with respect to the variable $x_{2}$ with the period εT. It means that $a_{ijkl}(x)$ are the high-oscillating functions.

**Figure 3:**
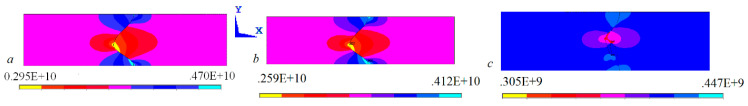
The local von Mises stresses corresponding to the tension along Ox-axis for (**a**) symmetric wave, (**b**) asymmetric wave, (**c**) wave with crest, Figure 3c.

**Figure 4:**
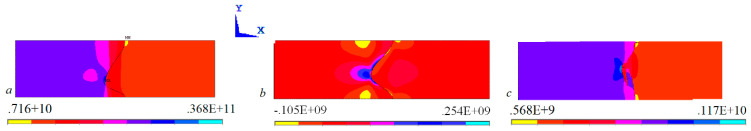
The local von Mises stresses corresponding to the tension along *Oy*-axis for (**a**) symmetric wave, (**b**) asymmetric wave, (**c**) wave with crest, Figure 3c.

**Figure 5:**
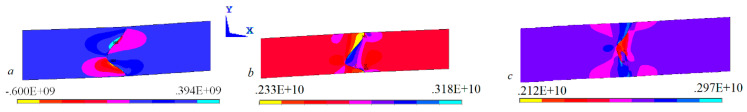
The local von Mises stresses corresponding to the shift for (**a**) symmetric wave, (**b**) asymmetric wave, (**c**) wave with crest, Figure 3c.

If welded bodies are subjected to a macroscopic force, at the macro-level, boundary layers [27,28,29] arise on the left and on the right of the weld D. At the micro-level, the weld D turns into a weld zone $D_{\varepsilon }$ and, in addition to the “left” and the “right” boundary layers, the local deformation occurs in the weld zone $D_{\varepsilon }$ placed between the “left” and the “right” boundary layers. The local stress–strain state inside the weld zone $D_{\varepsilon }$ is of general form (see Figure 3, Figure 4 and Figure 5 below) and demonstrates no similarity with the boundary layers. The boundary layers have classical form at a distance from the weld zone $D_{\varepsilon }$, in the domains between the weld zone $D_{\varepsilon }$ and the main parts $P^{-}$ and $P^{+}$ of the connected bodies. The local stress–strain state in the weld zone $D_{\varepsilon }$ is a micro-structural solution, while the boundary layers are macro-structural solutions.

Following [18,19], we seek the solution to the problem in the form:

(3) $$u(x)=u_{0}(x)+\varepsilon u_{1}(x/\varepsilon ).$$

In (3), $u_{0}(x)$ is the solution to the macroscopic problem (see Figure 2a,b):

(4) $$\left\{ \begin{array}{l} {\left(a_{ijkl}(x)u_{0k,ly}\right)}_{,iy}=q_{i}(x)\;\mathrm{in}\;P_{0},\\ u_{0}(y_{1}-0,y_{2})=u_{0}(y_{1}+0,y_{2})\;\mathrm{on}\;D,\;\\ \sigma_{n}(y_{1}-0,y_{2})=\sigma_{n}(y_{1}+0,y_{2})\;\mathrm{on}\;D,\\ \;u_{0}(x)=0\;\mathrm{on}\;\partial Q\; \end{array}\right.$$

and corrector $\varepsilon u_{1}(x/\varepsilon )$ is the “local perturbations”, i.e., $\varepsilon u_{1}(x/\varepsilon )$ satisfied the following conditions:

(5) $$u_{1}(y)\to 0\;\mathrm{as}\;|y_{3}|\to \infty \;\mathrm{and}\;u_{1}(y)\to 0\;\mathrm{is}\;\mathrm{periodic}\;\mathrm{in}\;(y_{1},y_{2})\;\mathrm{with}\;\mathrm{PC}\;T$$

The local perturbation

- decays in $y_{3}$ with distance from the weld $D_{\varepsilon }$;
- oscillates in the variables $\left(y_{1},y_{2}\right)$ with PC T.

Substituting equality (3) into the elasticity theory equations, we arrive at the following periodicity cell problem (see for details [18,19]):

(6) $$\left\{ \begin{array}{l} {\left(a_{ijkl}(y)u_{1k,ly}+a_{ij\alpha \beta }(y)u_{0\alpha ,\beta x}^{}(x)\right)}_{,iy}=0\;\mathrm{in}\;P_{0},\\ u_{1}(y)\;\mathrm{is}\;\mathrm{periodic}\;\mathrm{in}\;y_{1},y_{2}\;\mathrm{with}\;\mathrm{PC}\;T,\;\\ u_{1}(y)\to 0\;\mathrm{as}\;|y_{3}|\to \infty \end{array}\right.$$

In (6), the local elastic constant is given by (2).

**Figure 6:**
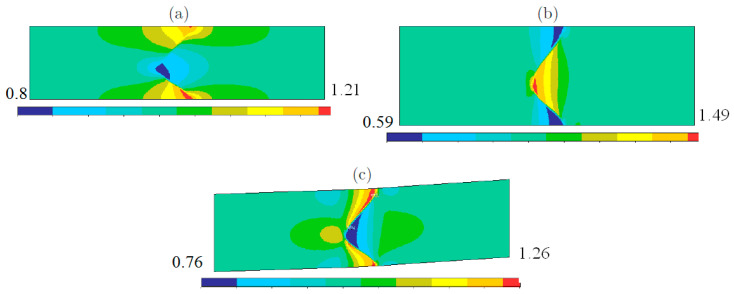
The von Mises stress concentration in the vicinity of symmetric weld: (**a**) tension along Ox-axis, (**b**) tension along Oy-axis, (**c**) shift.

**Figure 7:**
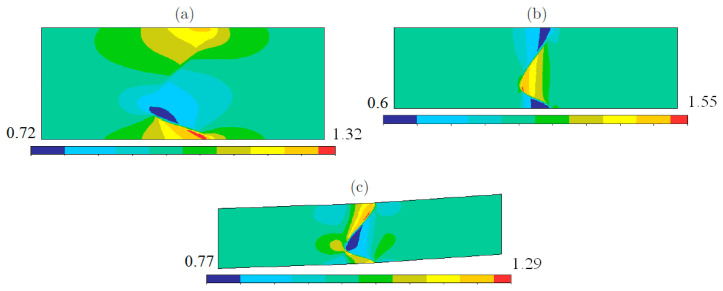
The von Mises stress concentration in the vicinity of asymmetric weld: (**a**) tension along Ox-axis, (**b**) tension along Oy-axis, (**c**) shift.

Problem (6) looks similar to the periodicity cell problem of the homogenization theory [20]. The difference is the decay condition: $u_{1}(y)\to 0$ as $|y_{3}|\to \infty$ in (6), while in the homogenization theory, the solution to the periodicity cell problem is periodic in all the variables [20]. This difference leads to significant differences between the limit problems. The first one is that the individual properties of the weld do not influence the macroscopic conditions on the weld surface, see for details [18,19]. The second difference is the discontinuity of several components of the macroscopic stress–strain state at the weld surface (the term $u_{0\alpha ,\beta x}^{}(x)$ in (6) corresponds to the macroscopic stress–strain state). The “jump” of macroscopic deformations is seen in Figure 5a, Figure 6c and Figure 7c. As a result of this “jump”, separation of the “fast” and the “slow” variables becomes impossible. The essence of the separation of the “fast” and the “slow” variables is treating $u_{0\alpha ,\beta x}^{}(x)$ as a parameter and “removing” it from the problem (6). This separation of the “fast” and the “slow” variables is the basis for the classical homogenization theory technique and leads to the periodicity cell problem arising in the homogenization theory [20]. In the case under consideration, we have to solve problem (6) in the original form. Note that a compatibility condition arises for the problem (6), see [18,19] for details. The existence of the compatibility condition also distinguishes (6) from the periodicity cell problem of the homogenization theory (it is known, see, e.g., [20], that there is no compatibility condition for the periodicity cell problem of the homogenization theory).

## 3. Numerical Computation of the Local Stresses in the Vicinity of the Waved Weld

As above, s,t mark the tangential and n marks the normal direction in the coordinates related to the welding seam. In Figure 1, $\left(s,t,n)=(x_{1},x_{2},x_{3}\right)$.

It is important for the following that at the macroscopic interface surface D, see Figure 2c, the strains $e_{ss},\;e_{tt},\;e_{st}$ and stresses $\sigma_{nn},\;\sigma_{ns},\;\sigma_{nt}$ are continuous [22]. The remaining strains and stresses may be discontinuous [22].

The total number of the continuous components of the macroscopic stress–strain state is six, as the number of stress (or strain) tensor components. Thus, one can express the microscopic stresses in the weld zone through the six continuous components $e_{ss},\;e_{tt},\;e_{st}$ and $\sigma_{nn},\;\sigma_{ns},\;\sigma_{nt}$ of the macroscopic stress–strain tensors.

By using Hooke’s law and Formula (3), we write the local (microscopic) stresses $\sigma_{ij}^{loc}(x)$ in the following form:

(7) $$\sigma_{ij}^{loc}(x)=a_{ijkl}(x/\varepsilon ){\left(u_{0}^{}(x)+\varepsilon u_{1}(x,x/\varepsilon )\right)}_{k,ly}\approx a_{ijkl}(x/\varepsilon )u_{1k,ly}+a_{ij\alpha \beta }(x/\varepsilon )u_{0\alpha ,\beta x}^{}(x).$$

As follows from [18,19], one can write the Formula (7) in the terms of the six continuous components of the macroscopic stress and strain tensors ($\left\{\sigma_{nt}\}=\{\sigma_{33},\;\sigma_{31},\;\sigma_{32}\right\}$ and $\left\{e_{st}\}=\{e_{11},\;e_{22},\;e_{12}\right\}$) in the form:

(8) $$\sigma_{ij}^{loc}(x)=K_{ij}^{nn}(x/\varepsilon )\sigma_{nn}(x)+K_{ij}^{nt}(x/\varepsilon )\sigma_{nt}(x)+K_{ij}^{st}(x/\varepsilon )e_{st}(x),$$

where $K_{ij}^{nn}(y)$ and $K_{ij}^{nt}(y)$ are the functions similar to the local stress concentration tensors [30,31], $K_{ij}^{st}(y)$ are stresses in problem (6) with unit macroscopic strains. All these functions are expressed through the elastic stresses in (6).

Consider the planar problem. An argument in favor of such consideration is the geometry of the explosion weld. The geometry of the explosion weld changes significantly in two directions and changes slowly in the third direction. If we neglect slow changes in the third direction, we arrive at a problem that depends on two spatial variables. Such a three-dimensional problem of elasticity theory can be reduced to a planar problem. The planar periodicity cell problem corresponds to the planar strain state (i.e., the local strains $\varepsilon_{xz}^{}(y)=\varepsilon_{yz}^{}(y)=0$ and $\varepsilon_{zz}^{}(y)=0$). The local stresses $\sigma_{xz}^{}(y)=\sigma_{yz}^{}(y)=0$ and $\sigma_{zz}^{}(y)=\nu (y)(\sigma_{xx}^{}(y)+\sigma_{yy}^{}(y))$.

Following, we present the result of our numerical calculations. The problem (6) was solved using the FEM program ANSYS [32]. In the computations, the welded material was as follows: on the left, there was copper: Young’s modulus $E=1\cdot 10^{11}$ Pa, Poisson’s ratio ν = 0.35, tensile strength $\sigma_{Cu}^{0}=180\times 10^{6}$ Pa; on the right there was aluminum: Young’s modulus $E=0.5\cdot 10^{11}$, Poisson’s ratio ν = 0.34, tensile strength $\sigma_{Al}^{0}=100\times 10^{6}$ Pa. We carried out the numerical computations for the three typical shapes of the weld wave: symmetric sinusoidal-like wave (Figure 1a), asymmetric wave (Figure 1b) and wave with a crest (Figure 1c). The weld geometries were constructed based on the measurement of the photograph shown in Figure 1a–c. The computations were done for three basic planar strains: the tensions along Ox- and Oy-axes and the shift in Oxy-plane. Note that not all of these strains were continuous.

*Tension along*Ox*-axis*. We considered three widely observed experimental types of weld waves: symmetric wave, Figure 2a, asymmetric wave, Figure 2b, and wave with crest, Figure 3c. The results of the numerical computations for the three types of weld waves subjected to the tension along Ox-axis are presented in Figure 3. Figure 3 displays the actual von Mises stress in the periodicity cells.

*Tension along*Oy*-axis*. Figure 4 displays the actual von Mises stress in the periodicity cell for three types of the weld waves.

*Shift in*Oxy*-plane*. Figure 5 displays the actual von Mises stress in the periodicity cell for three types of the weld waves.

## 4. The Problem of the Weld Strength

We assumed that the strength criteria for the connected materials had the form $f_{i}(\sigma_{ij}^{loc})<\sigma_{i}^{*}$ (the index i being the index of the material). After the problem (6) was solved, one could verify the strength criterion at every point of the connected materials. This approach correlated with the idea of the homogenization theory and with the approach used in the sub-structural analysis [33,34].

Two approaches (densely related one to another) are possible. The first is the computation of the local stresses in the vicinity of the weld, and the following fracture analysis. This is the problem related to the geometry of the weld. This approach is densely connected with the idea of the local stress concentration in inhomogeneous materials and structures [35]. The explosion not only forms a weld of complex geometry, but also changes the properties of the welded materials. It is possible to separate, at least theoretically, the problems related to the geometry of the weld and the problems of material properties in the weld. The second approach assumes the construction of the so-called homogenized strength criterion (if it exists for the problem under consideration).

We present solutions to the mentioned two problems for the planar problem, where the numerical computations are not as massive as in three-dimensional problems. In the planar problems, the interface strength criterion depends on following three macroscopic continuous stress–strain components: the macroscopic stresses $\sigma_{nn},\;\sigma_{ns}$ and the macroscopic strain $\varepsilon_{ss}$. In both approaches, it is necessary to calculate the functions, $K_{ij}^{nn}(y)$, $K_{ij}^{n3}(y)$ and $K_{ij}^{33}(y)$ (the direction normal to the weld is $x_{3}$ − n).

The functions $K_{ij}^{nn}(y)$, $K_{ij}^{n3}(y)$ and $K_{ij}^{33}(y)$ (i,j=1,2) were obtained by dividing the stresses $\sigma_{ij}^{nn}(y)$, $\sigma_{ij}^{n3}(y)$, $\sigma_{ij}^{33}(y)$ by the macroscopic stresses (for $\sigma_{ij}^{nn}(y)$ and $\sigma_{ij}^{n3}(y)$) or macroscopic strain (for $\sigma_{ij}^{33}(y)$) at the long distance from the weld. At the distance “far from the weld”, the microscopic stresses/strains coincide with macroscopic stresses/strains. In accordance with our computations, the “far from the weld” distance was 3–5 widths of PC. Thus, all the data necessary for computation $K_{ij}^{nn}(y)$, $K_{ij}^{n3}(y)$ and $K_{ij}^{33}(y)$ are determined from the solution to the problem (6). Note that, although the microscopic and macroscopic stresses/strains coincide, the values may be different on the left and on the right from the weld. For this reason, $K_{ij}^{n3}(y)$ are not exactly the classical stress concentration tensors.

### 4.1. Stress Concentrations in the Weld Zone

When the periodicity cell is subjected to tension along Ox-axis or shift, the stresses to the left and right of the weld are continuous and the macroscopic deformations are discontinuous. When the periodicity cell is subjected to tension along the Oy-axis, the stresses to the left and right of the weld are discontinuous and the macroscopic deformation is continuous. This situation shows a similarity with the Voigt- and Reiss-type deformations arising in a layered material. In the case under consideration, the deformations in the materials were localized near the weld. In the case of macroscopic tension along Ox-axis or shift, the von Mises stress concentration in the weld zone is calculated according to the formula:

$$k(x)=\sigma_{M}(x)/\sigma_{M}(\infty ),$$

In the case of the macroscopic tension along the Oy-axis, the von Mises stress concentration is determined as follows:

- to the left of the weld, $k(x)=\sigma_{M}(x)/\sigma_{M}{|}_{Z_{1}=-\infty }$;
- to the right of the weld, $k(x)=\sigma_{M}(x)/\sigma_{M}{|}_{Z_{1}=+\infty }$.

Here $\sigma_{M}$ means the stress intensity, and $\sigma_{M}{|}_{Z_{1}=\pm \infty }$ are stresses to the left and right of the weld, at the distances “far from the weld”. According to the results of numerical calculations from [17], these distances are (3−5)T (T is the characteristic dimension of the weld waves in the “fast” variables, see Figure 1).

The maximum Mises stress concentration is determined as $k=\underset{x}{\max}\;k(x)$.

Following, we present the results of our numerical computations. Figure 6 displays the von Mises stress concentrations in the periodicity cell corresponding to the symmetric weld.

Figure 7 displays the von Mises stress concentrations in the periodicity cell corresponding to the asymmetric weld.

**Figure 8:**
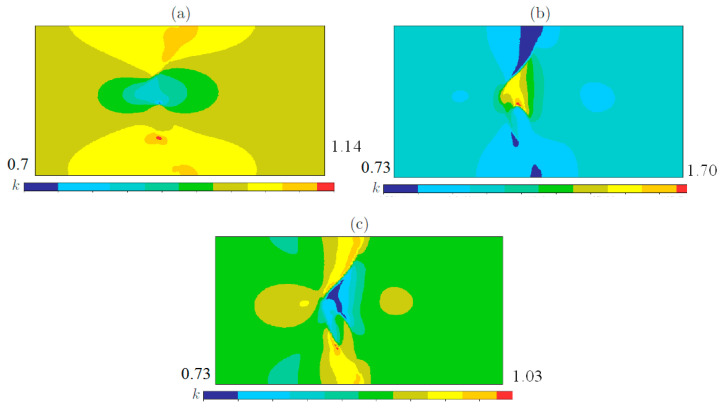
The von Mises stress concentration in the vicinity of the weld with crest: (**a**) tension along Ox-axis, (**b**) tension along Oy-axis, (**c**) shift.

Figure 8 displays the von Mises stress concentrations in the periodicity cell corresponding to the weld with crest.

We collected the maximum computed von Mises stress concentrations in Table 1.

### 4.2. Constructing the Macroscopic Strength Criterion of the Weld

In this section, we discuss the following question: “Is the weld an independent object with its own strength characteristics, or is it a sub-structural fragment of its encompassing structure without its own physical properties?” Note that in the classical elasticity theory, the interface does not form an independent object. Although the interface conditions in the classical elasticity theory are written in terms of the characteristics of the connected bodies [22], the interface is not a sub-structural fragment of the connected bodies.

In this section, we present an answer to the question under the assumption that the local strength limits are constant over the connected material. This assumption does not take into account the phenomenon of strain hardening. We discuss accounting for the strain hardening below. Note that the analysis presented in the previous section provided us with the stress concentration coefficients in the weld and did not involve the strength of the materials.

Let the strength criteria of the welded materials have the form

$$f^{-}(\sigma_{ij}^{})<\sigma_{}^{*-}(y),\;f^{+}(\sigma_{ij}^{})<\sigma_{}^{*+}(y)$$

(“−” marks the material to the left of the weld, “+” marks the material to the right of the weld).

The strength criterion in the arbitrary point y may be written in the form:

(9) $$f(y,\sigma_{ij}^{loc}(y))<\sigma_{}^{*}(y)$$

where the local stress limit is:

(10) $$\sigma^{*}(y)=\left\{ \begin{array}{l} \sigma^{*-}\;\mathrm{in}\;P^{-},\\ \sigma^{*+}\;\mathrm{in}\;P^{+} \end{array}\right.$$

and the function is:

$$f(y,\sigma_{ij}^{})=\left\{ \begin{array}{l} f^{-}(\sigma_{ij}^{})\;\mathrm{in}\;P^{-},\\ f^{+}(\sigma_{ij}^{})\;\mathrm{in}\;P^{+} \end{array}\right.$$

Substituting $\sigma_{ij}^{loc}(y)$ following (8) into (9), we obtain:

(11) $$f(y,K_{ij}^{nt}(x/\varepsilon )\sigma_{nt}(x)+K_{ij}^{st}(x/\varepsilon )e_{st}(x))<\sigma_{}^{*}(y)$$

As explained in [35], there is no failure at any points of the PC if the following inequality is satisfied:

(12) $$F(\sigma_{nt}(x),e_{st}(x))\equiv \underset{y\in P}{\max}\frac{f(x/\varepsilon ,K_{ij}^{nt}(x/\varepsilon )\sigma_{nt}(x)+K_{ij}^{st}(x/\varepsilon )e_{st}(x))}{\sigma_{}^{*}(y)}<1.$$

The failure starts when the condition $F(\sigma_{nt}(x),e_{st}(x))=1$ is satisfied. The failure occurs at the points $y_{0}$ at which:

(13) $$f(y_{0},K_{ij}^{nt}(y_{0})\sigma_{nt}(x)+K_{ij}^{st}(y_{0})e_{st}(x))=\sigma_{}^{*}(y_{0}).$$

Both the “no failure” criteria (12) and the “first failure” condition $F(\sigma_{nt}(x),e_{st}(x))=1$ are written in terms of macroscopic strains $e_{ss},\;e_{tt},\;e_{st}$ and macroscopic stresses $\sigma_{nn},\;\sigma_{ns},\;\sigma_{nt}$. This means that the “no failure” and the “first failure” criteria at the micro-level can be written in terms of the macroscopic stress–strain state.

When considering a planar problem, the macroscopic strength criterion of the interface can be written in the terms of two macroscopic stresses $\sigma_{nn},\;\sigma_{nt}$ and one strain $\varepsilon_{tt}$. It has the form $F(\sigma_{nt}(x),e_{st}(x))=1$.

In our computations, we used the von Mises strength criterion at the micro-level. This strength criterion is often used for plastic materials, in particular, metals (see, e.g., [36]). Then $f(y,\sigma_{ij}^{loc}(y))=\sigma_{Mises}^{loc}(y)$.

The macroscopic strength criterion of the weld can be constructed in the following steps:

1. Compute $K_{ij}^{nn}(y)$, $K_{ij}^{n3}(y)$ and $K_{ij}^{33}(y)$, and save the computed values.

2. Form the linear combinations:

(14) $$\sigma_{ij}(y)=K_{ij}^{33}(y)\varepsilon_{xx}+K_{ij}^{nn}(y)\sigma_{yy}+K_{ij}^{n3}(y)\sigma_{xy}.$$

and select from the combinations in (14) ones for which the condition in (15) is satisfied:

(15) $$\underset{y\in P}{\max}\frac{\sigma_{Mises}^{}(y)}{\sigma_{}^{*}(y)}=1\;(\mathrm{or}\;\leq 1).$$

In (15), $\sigma_{Mises}^{}(y)$ means von Mises stress corresponding to the local stresses (14).

Step 1 may be done with ANSYS or any similar FEM software. Step 2 requires additional programming. The program must process the ANSYS data files and make the computations indicated in (14) and (15).

The coefficients of the linear combinations (14) satisfying equality (15) form the macroscopic failure surface of the weld.

One can make calculations (14) and (15) in each of the welded bodies: in the left domain $p_{-}$ and in the right domain $p_{+}$ (see Figure 1c) separately and compute $\sigma_{}^{-}=\underset{y\in P_{-}}{\max}\frac{\sigma_{Mises}^{loc}(y)}{\sigma_{}^{*}(y)}$ and $\sigma_{}^{+}=\underset{y\in P_{+}}{\max}\frac{\sigma_{Mises}^{loc}(y)}{\sigma_{}^{*}(y)}$. The strength criterion (15) may be written in the form $\max(\sigma_{Mises}^{-},\sigma_{Mises}^{+})\leq 1$. The failure starts at $\max(\sigma_{Mises}^{-},\sigma_{Mises}^{+})=1$. This equality means $\sigma_{}^{-}=1$ or $\sigma_{}^{+}=1$. Thus, it is possible to indicate in which materials the failure starts (in other words, it is possible to indicate the “weakest” material).

The authors performed computations described in item 1 by using ANSYS FEM software and developed a computer program in order to perform the computations mentioned in item 2.

*Biaxial tension*. Consider the case of biaxial tension. In this case the macroscopic shift stress $\sigma_{12}=0$, and the strength criterion of the weld is written in terms of the continuous macroscopic axial tension $\varepsilon_{22}$ and axial stress $\sigma_{11}$. The failure surface is a curve in plane $O\sigma_{xx}\varepsilon_{yy}$. It is suitable to express the strength criteria of the welded materials, aluminum and copper, also in the terms of $\sigma_{11}$ and $\varepsilon_{22}$. The domains $S_{Al}=${$\left(\sigma_{11},\varepsilon_{22}\right)$: $\sigma_{Mises}^{}(\sigma_{11},e_{22})\leq \sigma_{Al}^{*}$} and $S_{Cu}=$$\sigma_{Mises}^{}(\sigma_{11},e_{22})\leq \sigma_{Cu}^{*}$ display the failure surfaces of pure aluminum and copper, correspondingly. The domains $S_{-}=$ {$\left(\sigma_{11},\varepsilon_{22}\right)$: $\underset{y\in p_{-}}{\max\sigma_{Mises}^{loc}(y)/}\sigma_{Al}^{*}\leq 1$} and $S_{+}=${$\left(\sigma_{11},\varepsilon_{22}\right)$: $\underset{y\in p_{+}}{\max\sigma_{Mises}^{loc}(y)/}\sigma_{Cu}^{*}\leq 1$} display the copper and the aluminum failure surfaces in the vicinity of the weld (copper is to the left of the weld, aluminum is to the right of the weld).

**Figure 9:**
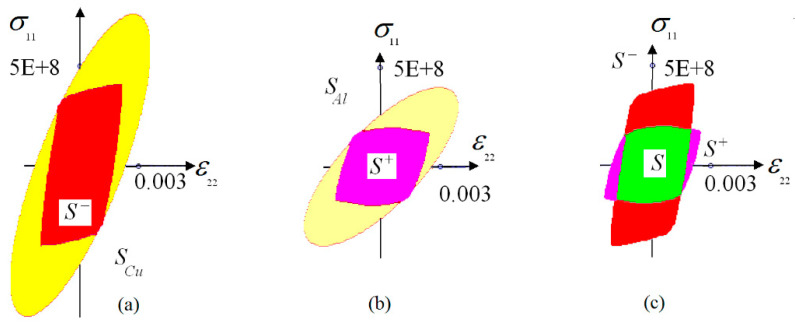
The failure curves for biaxial tension ((**a**) cooper, (**b**) aluminum, (**c**) weld).

The failure surface of the weld is the intersection $S=S_{-}\cap S_{+}$. We developed computer programs for the computation of the domains $S_{Cu}$, $S_{Al}$, $S_{-}$, $S_{+}$ and S. Figure 9 displays all the mentioned domains in the plane $O\sigma_{11}\varepsilon_{22}$.

It is seen in Figure 9a,b that aluminum and copper as the elements of the weld were less strong than the pure aluminum and copper, correspondingly. This was the result of the stress concentration in the weld zone, see Figure 6, Figure 7 and Figure 8. At the same time, we observed no direct relationship between the strength of materials in the weld zone and the strength of the pure materials. In our computations, copper was approximately twice as strong as the aluminum: $\sigma_{Cu}^{*}=180\times 10^{6}$ Pa and $\sigma_{Al}^{*}=100\times 10^{6}$ Pa. In the weld, on the top and the bottom of S, copper was the strongest element of the weld. On the left and the right sides of S, aluminum was the strongest element of the weld, see Figure 9c.

**Figure 10:**
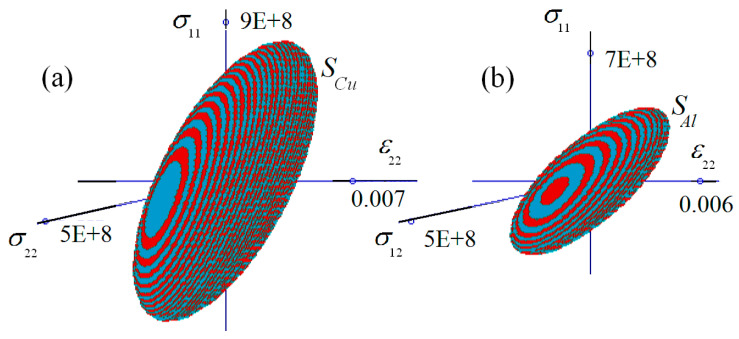
Failure surfaces: (**a**) pure copper, (**b**) pure aluminum.

*Planar deformation of general form*. In the general case, $\sigma_{xx},\;\varepsilon_{yy},\;\sigma_{xy}$ take non-zero values, thus, the failure surfaces are three-dimensional objects in the coordinate system $O\sigma_{11}\varepsilon_{22}\sigma_{12}$. Denote $S_{Al}=${$\left(\sigma_{11},\varepsilon_{22},\sigma_{12}\right)$: $\sigma_{Mises}^{}(\sigma_{11},\varepsilon_{22},\sigma_{12})\leq \sigma_{Al}^{*}$}, $S_{Cu}=${$\left(\sigma_{11},\varepsilon_{22},\sigma_{12}\right)$: $\sigma_{Mises}^{}(\sigma_{11},\varepsilon_{22},\sigma_{12})\leq \sigma_{Cu}^{*}$}, $S^{-}=${$\left(\sigma_{11},\varepsilon_{22},\sigma_{12}\right)$: $\underset{y\in p_{+}}{\max}\sigma_{Mises}^{loc}(y)/\sigma_{Cu}^{*}\leq 1$} and $S^{+}=${$\left(\sigma_{11},\varepsilon_{22},\sigma_{12}\right)$: $\underset{y\in p_{-}}{\max}\sigma_{Mises}^{loc}(y)/\sigma_{Al}^{*}\leq 1$}. The domains $S_{Al}$ and $S_{Cu}$ are displayed in Figure 10. The surfaces are colored to improve the visibility of these objects.

**Figure 11:**
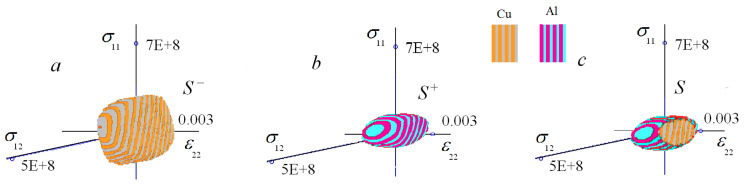
Failure surfaces: (**a**) for the left (copper) and (**c**) the right (aluminum) sides of the weld separately; (**b**) for the weld as a whole, (**a**,**b**) $S^{-}$ and $S^{+}$ together.

The domains $S^{-}$ and $S^{+}$ are displayed in Figure 11a,b. The weld failure surface S is the intersection of $S^{-}$ and $S^{+}$: $S=S^{-}\cap S^{+}$.

In the case under consideration, a large part of $S^{-}$ lay inside $S^{+}$, but not all $S^{-}$. The domain $S^{-}$ cut off the left and right parts of $S^{+}$. The remaining part of $S^{-}$ was the $S=S^{-}\cap S^{+}$. It is displayed in Figure 11c. The failure started on the left (copper) or the right (aluminum) side of the weld. It depended on the macroscopic stress–strain state of the weld.

The failure surfaces $S_{Al}$ and $S_{Cu}$ are compared in Figure 10 and the weld failure surface $S=S^{-}\cap S^{+}$ in Figure 11b. This comparison made it possible to estimate the decrease in the strength of the weld compared with the strength of the welded materials numerically. In the case displayed in Figure 10 and Figure 11, the strength of the weld was between two to four times less than the strength of the welded materials.

## 5. Prospective

The effect of the microstructure on the mechanical properties of explosive welding joints is an actual problem nowadays, see, e.g., [37,38,39], and references in these papers. The problem requires both experimental and theoretical research. The number of experimental researches prevail over the number of theoretical researches. In the opinion of the authors, the two-scale method of local perturbations, developed in [18,19] and partially inspired by the homogenization method in composite material [20,27,28,29,30,31], is an adequate tool for both the theoretical analysis and numerical computation of the effect of the microstructure on the mechanical properties of explosive welding joints.

Above, we presented the theory of the two-scale method, as applied to weld joints, and demonstrated the capabilities of this method for some specific problems. Now, we discuss several prospective problems. The prospects for research in the field of strength of welds based on the analysis of the microscopic stress–strain state in them, by the methods of the mechanics of solids, are very diverse. Below, we discuss several prospective problems directly related to our research.

### 5.1. Accounting for Strain Hardening in a Weld

The photos of explosion weld in Figure 1 lead to the conclusion that during welding, significant deformations occur in the region of wave formation of the weld, and fade at a distance equal to one weld wave amplitude. Similar information may be found in [11,12,13,14].

The plastic deformations were non-zero in the weld zone and zero at a distance from the weld zone. This meant that the plastic deformations were functions of the spatial variable y. Correspondingly, the strength limit of the welded materials was the function of the spatial variable y and had the following form:

(16) $$\sigma^{*}(y)=\left\{ \begin{array}{l} \sigma^{*-}\;\mathrm{in}\;P^{-},\\ \sigma^{*0}(y)\;\mathrm{in}\;P_{{}^{0}},\\ \sigma^{*+}\;\mathrm{in}\;P^{+} \end{array}\right.$$

Replacing (10) with (16) does not bring a significant change to the mathematical algorithm described above. As for the stress concentrations, this replacement does not affect the calculations related to Section 4.2 at all.

The only problem is obtaining the function $\sigma^{*0}(y)$, which describes the strength of materials after strain hardening. If the function $\sigma^{*0}(y)$ is known, we arrive at the problem, which is reminiscent of the problems arising in connection with the graded materials discussed in [40,41,42,43]. One can construct an approximation of the function, $\sigma^{*0}(y)$, if one knows the characteristic magnitude of the plastic deformation during the welding process and the form of the functions in (16). Little is known about the exact values of these parameters. One can estimate the deformations in the weld zone by using the photographs of the weld zone. Regarding the form of the functions in (16), the papers on the graded material often assume that the distribution may be described by power functions, see, e.g., [40,41,42,43,44].

### 5.2. Accounting for Interlayer in the Weld Zone

**Figure 12:**
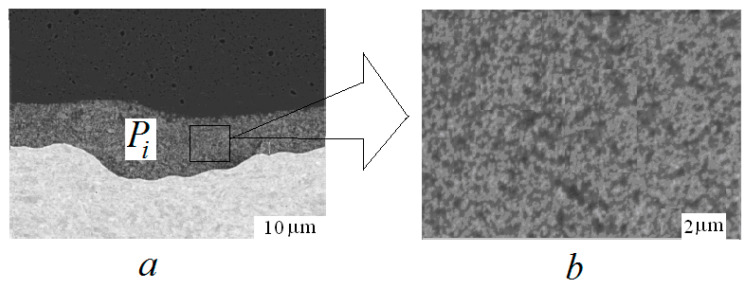
The interlayer in the weld zone melted zone ((**a**) view at the distance, (**b**) zoomed).

Between the welded metals there can be an interlayer which is both artificial (for example, an additional thin technological layer) and natural (for example, the “mixing zone” that occurs during the welding, Figure 12a).

This interlayer can be taken into account within the framework of the local perturbation approach if we introduce the interlayer region, $P_{i}$ and define the elastic constants and ultimate strength as follows:

(17) $$a_{ijkl}(y)=\left\{ \begin{array}{l} a_{ijkl}^{-}\;\mathrm{in}\;P^{-},\\ a_{ijkl}^{}\;\mathrm{in}\;P_{i},\\ a_{ijkl}^{+}\;\mathrm{in}\;P^{+}. \end{array}\right.\sigma^{*}(y)=\left\{ \begin{array}{l} \sigma^{*-}(y)\;\mathrm{in}\;P^{-},\\ \sigma^{*i}\;\mathrm{in}\;P_{i},\\ \sigma^{*+}(y)\;\mathrm{in}\;P^{+} \end{array}\right.,$$

The layer shown in Figure 12b looks like a typical composite material of random structure. So, then you can use the homogenization method to calculate (estimate) the elasticity constants $a_{ijkl}^{}\;\mathrm{in}\;P_{i}$ and the strength limit $\sigma^{*i}\;\mathrm{in}\;P_{i}$. Undoubtedly, this is a non-trivial problem, since the material in the domain $P_{i}$ has a random structure and its components subjected to large plastic deformations. However, such problems are being studied, and some results can be found in the literature on composite materials, see [45,46] for homogenization of random composites and [47] for homogenization in a plastic problem, see also reference in [45,46].

## 6. Conclusions

We presented the theory of the two-scale method, as applied to weld joints, and used this method for construction of the strength criterion of the joint. We applied the developed method to the computation of the local stress concentrations in the explosion weld. In accordance with our computation, the stress concentration in the explosion welding zone changed from 1.2 to 1.7, depending on the weld wave geometry and the macroscopic deformation. If strain hardening exceeded stress concentration, the strength of the explosion weld metals exceeded the strength of the weakest material of the pair and destruction occurred at a certain distance from the plane. In accordance with our considerations, the destruction did not necessarily occur along the least durable metal. In accordance with our considerations, the weakest point is determined not by the strength limit (the strength limit after the strain hardening), but by the ratio:

$$\frac{\mathrm{strength}\;\mathrm{limit}\;\mathrm{after}\;\mathrm{the}\;\mathrm{strain}\;\mathrm{hardening}}{\mathrm{stress}\;\mathrm{concentration}\;\mathrm{ratio}}$$

At the same time, if the strain hardening in the weld was small. compared with the stress concentration in the weld, the destruction occurred along the weld.

In any case, at the macrolevel, the weld may be treated as an independent solid mechanical object, possessing its own macroscopic strength criterion.

## Figures and Tables

**Table 1:** Maximum von Mises stress concentration k for different welds and different macroscopic strains.

Weld Type	*k*		
	Tension along the *Ox*-Axis	Tension along the *Oy*-Axis	Shift in the *Oxy*-Plane
Symmetric wave	1.21	1.49	1.25
Asymmetric wave	1.31	1.54	1.28
Wave with a crest	1.14	1.70	1.20

## Data Availability

The data are available by request.
